# The Association of Fat Mass Deviation with Neonatal Morbidities in Preterm Infants

**DOI:** 10.3390/nu18132158

**Published:** 2026-07-03

**Authors:** Maria Lithoxopoulou, Dimitrios Rallis, Anastasia Gkampeta, Eftychia Drogouti, Konstantina Kapetaniou, Helen Christou, Christos Tsakalidis

**Affiliations:** 1 Second Department of Neonatology, School of Medicine, Faculty of Health Sciences, Aristotle University of Thessaloniki, 541 24 Thessaloniki, Greece; drallis@auth.gr (D.R.); anastagab@yahoo.gr (A.G.); edrogouti@yahoo.com (E.D.); tsakalidisx@gmail.com (C.T.); 2 Fourth Department of Pediatrics, School of Medicine, Faculty of Health Sciences, Aristotle University of Thessaloniki, 541 24 Thessaloniki, Greece; kapetaniouk@gmail.com; 3 Brigham and Women’s Hospital, Harvard Medical School, Boston, MA 02115, USA; helen.christou@childrens.harvard.edu

**Keywords:** bioelectrical impedance analysis, body composition, fat mass, prematurity

## Abstract

**Background/Objectives:** Evidence suggests that assessment of neonatal body fat mass (FM) may provide additional insight into the relationship between disrupted fetal growth and neonatal health outcomes. Our objective was to determine whether neonatal body composition parameters are associated with hypoglycemia, hypothermia, and respiratory distress syndrome. **Methods:** We conducted a prospective study of preterm neonates born at ≤37 weeks of gestational age. Body composition was measured in the first week of life with bioelectrical impedance analysis (BIA). **Results:** We studied 101 neonates of mean gestational age 32.2 ± 3.2 weeks and mean birth weight 1792 ± 706 g. Neonates with intrauterine growth restriction (IUGR) had a significantly lower FM percentage (6.8 ± 3.6 compared with 8.3 ± 3.6%, *p* = 0.038), and higher free-FM percentage (93.1 ± 3.6 compared with 91.6 ± 3.4%, *p* = 0.041), compared with non-IUGR neonates. Compared with neonates with normal FM, neonates with decreased FM presented higher rates of hypothermia (35% compared with 9%, *p* = 0.005), hypoglycemia (46% compared with 9%, *p* < 0.001), and respiratory distress syndrome (58% compared with 27%, *p* = 0.008). Decreased FM percentage was significantly associated with hypoglycemia (OR 8.06, 95%CI 2.49–26.08, *p* < 0.001), hypothermia (OR 8.11, 95%CI 2.24–29.32, *p* = 0.001), and respiratory distress syndrome (OR 13.90, 95%CI 4.07–27.42, *p* < 0.001) after adjusting for gestational age, sex, and antenatal steroid administration. **Conclusions:** Lower FM percentage showed stronger associations with common neonatal morbidities, compared with standard anthropometric measurements alone. When a low FM percentage is detected, even when birth weight is normal, neonates at risk of undernutrition-related morbidity may benefit from closer nutritional and metabolic monitoring.

## 1. Introduction

Fetal growth restriction (FGR), clinically commonly referred to as intrauterine growth restriction (IUGR), describes impaired fetal growth resulting in failure to achieve genetically determined growth potential [1]. Small-for-gestational-age (SGA) neonates are defined by birth weight below the 10th percentile for gestational age [2,3]. Although the terms are often used interchangeably, important distinctions exist because some SGA neonates are constitutionally small and not growth-restricted [4].

The body composition of newborns born with growth restriction differs from that of neonates born with normal intrauterine growth [3]. Neonates receive glucose by oral consumption through breastfeeding or breast milk substitutes after delivery [5]. However, mobilization of body fat is crucial for the neonate before oral feeding is established because body fat provides alternative energy substrates for the glucose-dependent neonatal brain and other tissues. Neonates’ body fat percentage may be a reliable indicator of their risk of obtaining sufficient energy. Neonatal body fat percentage is determined by using several techniques, such as dual-energy X-ray absorptiometry, bioelectrical impedance analysis (BIA), and air displacement plethysmography [6]. The evidence on body composition in SGA neonates, especially among preterm neonates, is limited. Most studies assess body composition in term SGA neonates, leaving preterm SGA neonates under-represented [2,7]. Moreover, there is no consensus on standard methods or reference curves for body composition in preterm neonates, which hampers cross-study comparisons and limits understanding of early metabolic risk [2,7]. Nevertheless, limited evidence exists regarding the association between neonatal body fat percentage at birth and neonatal morbidity.

Thus, the study’s objectives were to examine the differences in body composition in a cohort of preterm neonates classified by population-based birth-weight percentiles, or IUGR, and to investigate whether nutritional classification based on body composition was more strongly associated with neonatal morbidities compared with anthropometric measurements alone.

## 2. Materials and Methods

We conducted a prospective cohort study including preterm neonates born at ≤37 weeks’ gestation who were admitted to our neonatal intensive care unit at Papageorgiou General Hospital, Thessaloniki, Greece, between January 2023 and June 2025. The study was approved by the Institutional Ethics Committee (Approval Number: 2021-Β2015-150), and an informed consent form was signed by each caregiver.

For all neonates of the study, we recorded the following maternal characteristics that were obtained from the maternal medical record: weight and height, which had been measured using the Seca Scale-up Line (Seca Medical Measuring Systems, Hamburg, Germany) in the first pregnancy visit and recorded in the medical records. Pre-pregnancy body mass index (BMI) was calculated using the formula: weight (Kg)/height^2^ (m^2^). Additionally, we recorded the existence of maternal pre-eclampsia, gestational diabetes, maternal smoking during pregnancy, the antenatal steroid administration (recorded as a full course), and the mode of delivery. Also, we recorded the neonatal characteristics, including the gestational age, birth weight [measured with the Seca 354 Scale (Seca Medical Measuring Systems, Hamburg, Germany)], birth length, and sex. Neonatal BMI was calculated from the formula: weight (Kg)/height^2^ (m^2^). The neonatal morbidities that were recorded included hypothermia occurring during the first 24 h of life, hypoglycemia occurring during the first 24 h of life, respiratory distress syndrome, patent ductus arteriosus, early- and late-onset sepsis, necrotizing enterocolitis, retinopathy of prematurity, intraventricular hemorrhage, and bronchopulmonary dysplasia (according to the Vermont–Oxford definitions) [3].

### 2.1. Body Composition

Body composition was assessed within the first week of life with Maltron BioScan touch i8-nano (Maltron International, Essex, UK). BIA has been validated in preterm neonates [8,9], while Maltron BioScan touch i8-nano has been calibrated for gestational age 23 weeks to 18 years and provides body composition analysis using tetra-polar BIA technology. The following parameters were evaluated: fat mass (FM), fat-free mass (FFM), total body water (TBW), intracellular water (ICW), extracellular water (ECW), body density, bone mineral density, dry weight, body surface area, body volume, extracellular mass, body cell mass, skeletal mass, total body mineral content, protein, calcium, glycogen, phase angle, impedance, resistance, and reactance.

Every neonate was measured in the morning. All measurements were performed in a thermoneutral environment (approximately 30 °C, humidity 50–60%) during the morning hours. Neonates were clinically stable and normothermic. Measurements were postponed in cases of crying, excessive movement, cardiorespiratory instability, or abnormal body temperature. To minimize the effect of rapid fluid shifts occurring during the first postnatal days, measurements were obtained under standardized clinical conditions and by the same trained investigators. The procedure lasted approximately 5 min and was performed 2 h after feeding to prevent emesis or interference with digestion when holding the neonate. During the exams, neonates were closely monitored to quickly identify any clinical changes that would compromise their health.

The neonates were placed in the chamber in a supine position without any clothing. The neonates were first weighed using an electronic scale that is a component of the device. The standardized test involved placing the external electrode (black emitter) on the third metacarpal bone, the internal arm electrode (red detector) on the dorsal surface of the right wrist between the ulnar and radial bones, the internal leg electrode on the anterior surface of the ankle between the prominent parts of the bones, and the external electrode on the surface of the third metatarsal bone. For this operation, a minimum of 5 cm separated the electrodes [10]. To prevent random dispersion of the electric current, neither the examiner nor the guardian touched the neonate throughout the tests, and the limbs were kept away from the body and metal surfaces.

### 2.2. Definitions

Birth weight for gestational age definition: Using the INTRGROWTH-21 weight for gestational age centiles, adjusted for sex, we classified each neonate as small-, appropriate-, or large-for-gestational age (SGA, AGA, or LGA, respectively) [11].

IUGR definition: IUGR was defined by the fetal abdominal circumference/estimated fetal weight, or the dopplers of the umbilical or uterine arteries, according to the International Society of Ultrasound in Obstetrics & Gynecology (ISUOG) guidelines [12].

Body fat definition: The FM percentage was evaluated for each neonate with BIA. Neonates with FM percentage ≤1 standard deviation (SD) below the cohort mean were classified as neonates with decreased FM. This SD-based threshold was chosen as an operational definition of relatively low neonatal adiposity, consistent with previous neonatal body composition studies evaluating relative nutritional depletion.

### 2.3. Statistical Analysis

Continuous variables were expressed as mean ± standard deviation or median (interquartile range), and categorical variables as *n* (percentage %). The normality of the distributions of continuous variables was assessed by the Kolmogorov–Smirnov or the Shapiro–Wilk test. Comparisons between continuous variables were performed with the Student’s *t*-test, or the non-parametric Mann–Whitney test, as appropriate, whereas comparisons between categorical variables were performed using the chi-square test or Fisher’s exact test. A subgroup analysis between neonates born at gestational age between 32 and 37 weeks and neonates born at gestational age less than 32 weeks was performed for the BIA parameters and the neonatal outcomes. A multivariate regression analysis was performed to separately examine the association of birth-weight deviation, IUGR, and FM percentage deviation with significant outcomes, adjusted for gestational age, sex, and antenatal steroid administration. The Bonferroni correction for multiple testing was utilized. Odds ratio (OR) and 95% confidence intervals (CI) were estimated.

All performed tests were two-sided, and a *p*-value less than 0.05 was considered statistically significant (α 0.05). Because the study was designed as a prospective observational cohort, including all eligible neonates during the study period, no formal a priori sample size calculation was performed. A post hoc power analysis using G*Power (version 3.1.9.7; [13]) demonstrated that the final sample of 101 neonates provided 80% power to detect a medium effect size (Cohen’s d = 0.57) between groups at a two-sided α level of 0.05. The data were analyzed using SPSS Statistics (IBM SPSS Statistics for Windows, Version 24.0. Armonk, NY, USA).

## 3. Results

### 3.1. Perinatal Characteristics

A total of 101 neonates with a mean gestational age of 32.2 ± 3.2 weeks and 1792 ± 706 g of birth weight was examined during the study period. The perinatal characteristics of the study cohort are presented in Table 1. Based on the INTERGROWTH-21 centiles, 79 (78%) neonates were AGA, and 22 (22%) neonates were SGA. Compared with AGA neonates, SGA neonates were of lower birth weight (1268 ± 462 compared with 1938 ± 695 g, *p* < 0.001), birth length (38.3 ± 5.6 compared with 43.1 ± 5.1 cm, *p* = 0.001), and BMI (7.7 ± 1.4 compared with 9.6 ± 2.6 Kg/m^2^, *p* = 0.003).

Moreover, 24 (24%) neonates of the total cohort were IUGR, while 77 (76%) were non-IUGR. IUGR neonates were of lower birth weight (1455 ± 640 compared with 1897 ± 696 g, *p* = 0.007), birth length (39.4 ± 6.1 compared with 42.8 ± 5.0 cm, *p* = 0.019), and BMI (8.2 ± 1.6 compared with 9.5 ± 2.7 Kg/m^2^, *p* = 0.026) compared with non-IUGR neonates.

Finally, according to BIA, 26 (26%) neonates of the total cohort were of decreased FM, and 75 (74%) of normal FM percentage. Neonates with decreased FM were of lower gestational age compared with neonates with normal FM (31.9 ± 2.4 compared with 33.3 ± 2.4 weeks, *p* = 0.024), and birth length (40.8 ± 5.8 compared with 45.1 ± 2.6 cm, *p* < 0.001), while 42% of neonates with decreased FM were IUGR (compared with 17% of neonates with normal FM, *p* = 0.016).

### 3.2. Bioelectrical Impedance Analysis

The parameters of BIA are presented in Table 2 and Table A1. SGA neonates had a significantly lower ECW percentage (62.6 ± 4.1 compared with 65.2 ± 4.4%, *p* = 0.017) and bone mineral density (0.20 ± 0.02 compared with 0.24 ± 0.03 g/cm^2^, *p* < 0.001) compared with AGA neonates. Also, IUGR neonates had a significantly lower FM percentage (6.8 ± 3.6 compared with 8.3 ± 3.6%, *p* = 0.038), higher FFM percentage (93.1 ± 3.6 compared with 91.6 ± 3.4%, *p* = 0.041), and lower bone mineral density (0.21 ± 0.03 compared with 0.24 ± 0.03 g/cm^2^, *p* = 0.002) compared with non-IUGR neonates. Finally, neonates with decreased FM, compared with those with normal FM percentage, had significantly lower FM percentage, ICW percentage, impedance, and resistance, while exhibiting higher FFM percentage, TBW percentage, ECW percentage, body density, body mineral density, and phase angle.

### 3.3. Neonatal Outcomes

The neonatal outcomes of the study cohort are presented in Table 3. No significant differences were recorded between SGA and AGA, or between IUGR and non-IUGR neonates in any of the measured neonatal outcomes. Neonates with decreased FM presented higher rates of hypothermia (35% compared with 9%, *p* = 0.005), hypoglycemia (46% compared with 9%, *p* < 0.001), and respiratory distress syndrome (58% compared with 27%, *p* = 0.008), as well as longer hospital stay [23 (14–50) compared with 17 (11–30) days, *p* = 0.025], compared with neonates with normal FM.

### 3.4. Subgroup Analysis

#### 3.4.1. Characteristics of Neonates Born Between 32 and 37 Weeks of Gestational Age

Among the total cohort, 52 neonates were born at gestational age between 32 and 37 weeks. The characteristics of the BIA are presented in Table A2, and the neonatal outcomes in Table A3. Neonates with decreased FM had a significantly lower FM percentage and higher FFM, TBW, and ECW percentages, compared with neonates with normal FM. Also, neonates with decreased FM presented with a higher rate of hypoglycemia and respiratory distress syndrome, compared with neonates with normal FM.

#### 3.4.2. Characteristics of Neonates Born Less than 32 Weeks of Gestational Age

Moreover, 49 neonates were born at a gestational age of less than 32 weeks. The characteristics of the BIA are presented in Table A4, and the neonatal outcomes in Table A5. Neonates with decreased FM had significantly lower FM percentage, ICW percentage, impedance, and resistance, while exhibiting higher FFM percentage, TBW percentage, ECW percentage, body density, body mineral density, and phase angle. Furthermore, neonates with decreased FM presented with a higher rate of hypothermia, hypoglycemia, and respiratory distress syndrome, as well as longer hospital stay, compared with neonates with normal FM.

### 3.5. Multivariate Analysis

In multivariate analysis, decreased FM percentage (FM < 1 SD below cohort mean) was significantly associated with hypoglycemia (OR 8.06, 95%CI 2.49–26.08, *p* < 0.001), hypothermia (OR 8.11, 95%CI 2.24–29.32, *p* = 0.001), and respiratory distress syndrome (OR 13.90, 95%CI 4.07–27.42, *p* < 0.001) after adjusting for gestational age, sex, and antenatal steroid administration (Table 4). Also, IUGR was significantly associated with hypoglycemia (OR 3.70, 95%CI 1.20–11.36, *p* = 0.022), and respiratory distress syndrome (OR 2.89, 95%CI 1.14–7.36, *p* = 0.025), after adjusting for the same variables (Table 4).

## 4. Discussion

The present prospective cohort study evaluated the association between body composition and common neonatal morbidities in preterm neonates using BIA during the first week of life. The principal findings were that preterm neonates with decreased FM percentage had significantly higher odds of hypoglycemia, hypothermia, and respiratory distress syndrome. The differences in BIA between neonates with decreased compared with neonates with normal FM percentage were more pronounced in the subgroup of neonates less than 32 weeks of gestational age. Furthermore, IUGR neonates exhibited lower FM percentages and higher FFM percentages than non-IUGR neonates, whereas differences between SGA and AGA neonates were less pronounced. These findings suggest that body composition assessment may provide information complementary to birth weight alone when evaluating nutritional status and metabolic vulnerability in preterm neonates.

Altered fetal growth is known to affect neonatal body composition [14,15]. While SGA and IUGR are often used interchangeably in clinical practice, they represent distinct entities [16]. SGA is an anthropometric classification based on birth-weight percentile, while IUGR reflects a pathological process characterized by impaired fetal growth [17,18]. Consequently, some SGA neonates are constitutionally small but otherwise healthy, whereas some neonates with IUGR may have birth weights within the normal range [19].

Our findings support this distinction. Although SGA neonates tended to have lower FM values than AGA neonates, the most pronounced differences were observed between IUGR and non-IUGR neonates. This observation suggests that FM percentage may better reflect fetal nutritional status than birth-weight classification alone. Similar findings have been reported in previous studies demonstrating that growth-restricted neonates exhibit reduced adiposity at birth despite variability in birth-weight percentiles [20,21]. A recent review that evaluated how IUGR affected the body composition of neonates during the first few months after birth discovered that neonates with IUGR had considerably lower FFM and FM than neonates without IUGR [2]. Several methodological differences should be considered when interpreting literature. First, many earlier studies included predominantly term SGA infants, by comparison our cohort consisted exclusively of preterm neonates, a population with distinct body composition dynamics and metabolic adaptation. Secondly, differences in the definitions of SGA and IUGR and the relatively small sample sizes of most neonatal studies may contribute to variability across published findings. Despite these methodological differences, the overall evidence consistently supports reduced fat mass as a marker of impaired fetal nutrition. Our study extends this knowledge by demonstrating that early reductions in fat mass are independently associated with clinically important neonatal morbidities, highlighting the potential value of body composition assessment beyond conventional anthropometric classification.

Similar results were reported in the systematic review by Manapurath et al., which demonstrated lower FM and FFM among neonates with IUGR [2]. Similarly, Verkauskiene et al. reported reduced FM in growth-restricted neonates, even among neonates classified as AGA by birth weight [22]. In contrast, we observed significant differences primarily in FM percentage, whereas changes in FFM were more modest. Several factors may explain this discrepancy, including differences in study populations, gestational age distribution, timing of assessment, and body composition methodology. Most notably, our cohort consisted exclusively of preterm neonates assessed during the first postnatal week using BIA, whereas many previous studies evaluated term neonates using air displacement plethysmography or dual-energy X-ray absorptiometry. Rapid postnatal changes in hydration status may additionally influence the estimation of FFM compartments during this period. Nevertheless, the consistent finding across studies is that impaired fetal growth is associated with altered body composition, particularly reduced adiposity [2,22].

One of the most important findings of the present study was the independent association between reduced FM percentage and common neonatal morbidities. Neonates with lower FM percentages demonstrated higher odds of hypoglycemia, hypothermia, and respiratory distress syndrome, even after adjustment for relevant clinical variables. The stronger associations observed among neonates born before 32 weeks suggest that body composition assessment may be particularly informative in very preterm populations, although these findings require confirmation in larger multicenter cohorts.

These observations are biologically plausible. Adipose tissue serves as an important energy reserve during the transition from intrauterine to extrauterine life. Reduced fat stores could be associated with lower substrate availability during periods of metabolic stress and may be associated with impaired glucose homeostasis [23]. Similarly, lower adiposity may be associated with reduced thermal insulation and energy availability for thermoregulation, potentially increasing susceptibility to hypothermia. Although the present observational design does not permit conclusions regarding causality, the observed associations are consistent with established physiological mechanisms linking nutritional reserves to neonatal adaptation.

The association between lower FM percentage and respiratory distress syndrome is also noteworthy. Previous studies have demonstrated that impaired fetal growth is associated with altered metabolic adaptation and increased neonatal morbidity [24,25,26,27,28,29,30]. In particular, neonates with impaired nutrition presented in higher rates of poor perinatal outcomes, including stillbirth, neonatal death, low Apgar scores [24], fetal distress [25,30], hypoglycemia, hypothermia [26,30], respiratory distress syndrome, intraventricular hemorrhage, necrotizing enterocolitis, longer hospital stay [27], and poor breastfeeding [28]. Even when the nutritional status was assessed with the clinical score CANSCORE, early newborn morbidities, such as hypoglycemia, feeding intolerance, and infection, were more common in neonates with impaired nutrition [31]. Reduced FM may therefore represent a marker of overall fetal nutritional compromise rather than a direct cause of respiratory disease. Future studies are required to determine whether body composition contributes independently to respiratory outcomes or primarily reflects underlying fetal growth disturbances.

Currently, neonatal nutritional assessment relies largely on anthropometric measurements such as birth weight, length, and head circumference. Although these measures remain clinically valuable [32], they provide limited information regarding body composition and energy reserves [22,33]. Collectively, these observations suggest that FM percentage identified neonates with greater vulnerability to metabolic complications despite apparently normal birth weight.

Notably, some neonates classified as AGA may exhibit reduced adiposity and therefore remain at risk for neonatal complications. Conversely, constitutionally small neonates may demonstrate relatively preserved body composition despite lower birth-weight percentiles. These observations support the concept that body composition assessment may complement conventional anthropometric evaluation and improve characterization of neonatal nutritional status.

The strengths of this study include its prospective design, standardized body composition assessment, and evaluation of multiple clinically relevant neonatal outcomes. In addition, body composition measurements were obtained during the early neonatal period using a non-invasive bedside technique that is feasible in routine clinical practice.

Several limitations should also be acknowledged. First, the study was conducted at a single center with a relatively modest sample size, which may limit generalizability. Second, it should be noted that decreased FM in the present study was defined as a value ≤1 standard deviation below the cohort mean. This threshold was derived from the study population and should not be considered a validated clinical cutoff. Nevertheless, it may serve as a preliminary indicator correlated with neonates at increased risk of metabolic instability. Such neonates may benefit from intensified glucose monitoring during the first 24–48 h of life, closer thermal surveillance, and individualized nutritional assessment. Future multicenter studies are required to establish clinically validated FM reference values and intervention thresholds. Moreover, although FM assessment may eventually contribute to individualized nutritional strategies, the present study did not evaluate body composition-guided interventions. Therefore, the potential benefits of incorporating FM measurements into routine clinical decision-making remain hypothetical and should be investigated in future interventional studies. Because of the observational design, the findings should be interpreted as associations rather than evidence of causality. Furthermore, we examined the body composition in preterm neonates solely within the first week of life, and thus, we could not examine the body composition longitudinally. However, BIA-derived body fat estimates may still be influenced by the rapidly changing hydration status of preterm neonates during the first week of life.

## 5. Conclusions

Reduced FM percentage was independently associated with higher odds of hypoglycemia, hypothermia, and respiratory distress syndrome in preterm neonates. Furthermore, IUGR neonates exhibited significantly altered body composition compared with non-IUGR neonates, supporting the concept that body composition assessment provides information beyond conventional birth-weight classification. Assessment of FM may represent a useful adjunct to anthropometric evaluation correlated with preterm neonates at increased risk of metabolic instability. Larger multicenter studies are required to establish reference values, validate clinically meaningful thresholds, and determine whether body composition-guided nutritional interventions improve neonatal outcomes.

## Figures and Tables

**Table 1:** Perinatal characteristics of the total and the subgroups of the cohort.

	Total Cohort (*n* = 101)	Appropriate-for-Gestational Age (*n* = 79)	Small-for-Gestational Age (*n* = 22)	*p*	No Intrauterine Growth Restriction (*n* = 77)	Intrauterine Growth Restriction (*n* = 24)	*p*	Normal Fat Mass% (*n* = 75)	Decreased Fat Mass% (*n* = 26)	*p*
Gestational age, weeks	32.2 ± 3.2	32.3 ± 3.2	32.0 ± 3.5	0.671	32.4 ± 3.1	31.6 ± 3.5	0.314	33.3 ± 2.4	31.9 ± 3.4	0.024
Birth weight, g	1792 ± 706	1938 ± 695	1268 ± 462	<0.001	1897 ± 696	1455 ± 640	0.007	1961 ± 545	1733 ± 748	0.102
Birth length, cm	41.9 ± 5.5	43.1 ± 5.0	38.3 ± 5.6	0.001	42.8 ± 5.0	39.4 ± 6.1	0.019	45.1 ± 2.6	40.8 ± 5.8	<0.001
Small-for-gestational age	22 (22%)	0 (0%)	22 (100%)	n/a	11 (14%)	11 (46%)	0.003	15 (20%)	7 (27%)	0.582
Intrauterine growth restriction	24 (24%)	13 (17%)	11 (50%)	0.003	0 (0%)	24 (100%)	n/a	13 (17%)	11 (42%)	0.016
Decreased fat mass percentage	26 (26%)	19 (24%)	7 (32%)	0.315	15 (20%)	11 (46%)	0.016	0 (0%)	26 (100%)	n/a
Body mass index, kg/m^2^	9.2 ± 2.5	9.6 ± 2.6	7.7 ± 1.4	0.003	9.5 ± 2.7	8.2 ± 1.6	0.026	9.0 ± 2.8	9.5 ± 1.5	0.388
Sex, male	53 (53%)	40 (51%)	13 (59%)	0.630	42 (46%)	13 (54%)	0.490	34 (45%)	19 (73%)	0.022
Maternal body mass index, kg/m^2^	25.1 ± 5.6	25.0 ± 5.5	25.5 ± 5.8	0.718	24.4 ± 5.6	27.4 ± 4.9	0.022	25.0 ± 5.9	25.6 ± 4.4	0.642
Maternal pre-eclampsia	13 (13%)	7 (9%)	6 (27%)	0.033	7 (9%)	6 (25%)	0.074	10 (13%)	3 (12%)	0.999
Maternal gestational diabetes	22 (22%)	21 (27%)	1 (5%)	0.038	18 (24%)	4 (17%)	0.581	16 (21%)	6 (23%)	0.999
Maternal smoking in pregnancy	14 (14%)	9 (11%)	5 (23%)	0.293	9 (12%)	5 (21%)	0.311	10 (13%)	4 (15%)	0.511
Antenatal steroid administration	75 (75%)	58 (63%)	17 (77%)	0.216	55 (71%)	21 (87%)	0.496	56 (75%)	19 (73%)	0.469
Delivery, cesarean section	95 (94%)	74 (94%)	21 (96%)	0.999	71 (92%)	24 (100%)	0.331	70 (93%)	25 (96%)	0.688

**Table 2:** Characteristics of the bioelectrical impedance analysis of the total and the subgroups of the cohort.

	Total Cohort (*n* = 101)	Appropriate-for-Gestational Age (*n* = 79)	Small-for-Gestational Age (*n* = 22)	*p*	No Intrauterine Growth Restriction (*n* = 77)	Intrauterine Growth Restriction (*n* = 24)	*p*	Normal Fat Mass% (*n* = 75)	Decreased Fat Mass% (*n* = 26)	*p*
Fat mass, %	8.0 ± 3.5	8.0 ± 3.5	7.7 ± 3.8	0.721	8.3 ± 3.6	6.8 ± 3.6	0.038	9.5 ± 2.7	3.4 ± 0.9	<0.001
Fat-free mass, %	91.9 ± 3.5	91.8 ± 3.4	92.1 ± 3.7	0.747	91.6 ± 3.4	93.1 ± 3.6	0.041	90.4 ± 2.7	96.3 ± 0.7	<0.001
Total body water, %	76.3 ± 4.6	76.4 ± 4.7	76.0 ± 4.5	0.715	76.1 ± 4.6	76.8 ± 4.8	0.558	74.2 ± 3.3	82.2 ± 2.1	<0.001
Intracellular water, %	35.8 ± 5.3	35.4 ± 5.5	37.3 ± 4.1	0.135	35.8 ± 5.4	35.7 ± 5.0	0.949	36.8 ± 3.6	32.9 ± 7.8	0.001
Extracellular water, %	64.9 ± 4.5	65.2 ± 4.4	62.6 ± 4.1	0.017	64.8 ± 4.3	64.2 ± 5.0	0.575	63.4 ± 4.1	68.4 ± 3.3	<0.001

**Table 3:** Neonatal outcomes of the total and the subgroups of the cohort.

	Total Cohort (*n* = 101)	Appropriate-for-Gestational Age (*n* = 79)	Small-for-Gestational Age (*n* = 22)	*p*	No Intrauterine Growth Restriction (*n* = 77)	Intrauterine Growth Restriction (*n* = 24)	*p*	Normal Fat Mass% (*n* = 75)	Decreased Fat Mass% (*n* = 26)	*p*
Hypothermia	16 (16%)	11 (14%)	5 (23%)	0.332	11 (14%)	5 (21%)	0.523	7 (9%)	9 (35%)	0.005
Hypoglycemia	19 (19%)	14 (18%)	5 (23%)	0.554	11 (14%)	8 (33%)	0.068	7 (9%)	12 (46%)	<0.001
Respiratory distress syndrome	35 (35%)	29 (37%)	6 (27%)	0.459	24 (31%)	11 (46%)	0.223	20 (27%)	15 (58%)	0.008
Late-onset sepsis	18 (18%)	12 (15%)	6 (27%)	0.355	13 (17%)	5 (21%)	0.999	16 (21%)	2 (7%)	0.127
Necrotizing enterocolitis	7 (7%)	3 (4%)	4 (18%)	0.058	5 (6%)	2 (8%)	0.999	7 (9%)	0 (0%)	0.179
Intraventricular hemorrhage	11 (11%)	7 (9%)	4 (18%)	0.249	7 (9%)	4 (17%)	0.452	10 (13%)	1 (4%)	0.281
Bronchopulmonary dysplasia	13 (13%)	9 (11%)	4 (18%)	0.472	9 (12%)	5 (21%)	0.728	12 (16%)	1 (4%)	0.175
Length of hospital stay, days	25 (15–50)	22 (13–44)	29 (17–54)	0.359	22 (13–44)	30 (16–54)	0.441	17 (11–30)	23 (14–50)	0.025

**Table 4:** Regression analysis of the association of hypoglycemia, hypothermia, and respiratory distress syndrome with body weight deviation, IUGR status, and fat mass percentage deviation, adjusted for gestational age, sex, and antenatal steroid administration.

	Hypoglycemia			Hypothermia			Respiratory Distress Syndrome		
	OR	95%CI	*p*	OR	95%CI	*p*	OR	95%CI	*p*
Body weight deviation	1.28	0.41–4.01	0.665	2.01	0.64–6.28	0.230	1.20	0.46–3.15	0.703
Gestational age	0.99	0.94–1.05	0.916	0.93	0.88–0.99	0.041	0.95	0.90–0.99	0.049
Sex, male	0.35	0.21–1.01	0.054	0.99	0.35–2.79	0.992	1.31	0.58–2.93	0.512
Antenatal steroids administration	0.84	0.48–1.47	0.553	0.70	0.38–1.26	0.242	1.22	0.75–1.99	0.407
Intrauterine growth restriction	3.70	1.20–11.36	0.022	1.84	0.58–5.81	0.296	2.89	1.14–7.36	0.025
Gestational age	0.97	0.92–1.02	0.265	0.94	0.89–0.99	0.048	0.92	0.88–0.97	0.003
Sex, male	0.27	0.08–0.84	0.024	0.92	0.32–2.63	0.881	1.15	0.50–2.65	0.734
Antenatal steroid administration	0.74	0.41–1.33	0.323	0.69	0.38–1.26	0.234	1.15	0.70–1.89	0.570
Decreased fat mass percentage	8.06	2.49–26.08	<0.001	8.11	2.24–29.32	0.001	13.90	4.07–27.42	<0.001
Gestational age	0.91	0.85–0.98	0.019	0.86	0.79–0.94	0.001	0.83	0.76–0.91	<0.001
Sex, male	0.45	0.15–1.39	0.168	1.44	0.48–4.35	0.512	2.33	0.89–6.11	0.085
Antenatal steroid administration	0.73	0.40–1.34	0.319	1.82	0.77–4.27	0.200	1.23	0.71–2.15	0.453

OR, odds ratio; CI, confidence intervals.

## Data Availability

The raw data supporting the conclusions of this article will be made available by the authors on reasonable request.

## Abbreviations

The following abbreviations are used in this manuscript:

IUGR	Intrauterine growth restriction
SGA	Small-for-gestational age
BIA	Bioelectrical impedance analysis
BMI	Body mass index
FM	Fat mass
FFM	Fat-free mass
TBW	Total body water
ICW	Intracellular water
ECW	Extracellular water
AGA	Appropriate-for-gestational age
LGA	Large for gestational age
ISUOG	International Society of Ultrasound in Obstetrics & Gynecology
OR	Odds ratio
CI	Confidence intervals

## Appendix A

**Table A1:** Characteristics of the bioelectrical impedance analysis of the total and the subgroups of the cohort.

	Total Cohort (*n* = 101)	Appropriate-for-Gestational Age (*n* = 79)	Small-for-Gestational Age (*n* = 22)	*p*	No Intrauterine Growth Restriction (*n* = 77)	Intrauterine Growth Restriction (*n* = 24)	*p*	Normal Fat Mass% (*n* = 75)	Decreased Fat Mass% (*n* = 26)	*p*
Body density, g/cm^3^	1.02 ± 0.01	1.02 ± 0.01	1.02 ± 0.01	0.852	1.02 ± 0.01	1.02 ± 0.01	0.851	1.01 ± 0.01	1.03 ± 0.01	<0.001
Bone mineral density, g/cm^2^	0.23 ± 0.03	0.24 ± 0.03	0.20 ± 0.02	<0.001	0.24 ± 0.03	0.21 ± 0.03	0.002	0.23 ± 0.03	0.25 ± 0.02	0.001
Phase angle, degrees	8.6 ± 2.7	8.4 ± 2.3	9.4 ± 3.7	0.231	8.5 ± 2.5	8.9 ± 3.3	0.577	8.2 ± 2.2	9.9 ± 3.6	0.033
Impedance, ohms	828 ± 229	808 ± 222	903 ± 246	0.087	820 ± 227	856 ± 239	0.509	906 ± 211	604 ± 91	<0.001
Resistance, ohms	798 ± 178	796 ± 179	805 ± 179	0.826	789 ± 174	827 ± 193	0.364	832 ± 178	698 ± 141	0.001
Reactance, ohms	124 ± 57	124 ± 61	120 ± 44	0.776	122 ± 60	130 ± 49	0.543	125 ± 62	118 ± 41	0.579
Body surface area, m^2^	0.14 ± 0.03	0.15 ± 0.03	0.12 ± 0.03	0.001	0.15 ± 0.03	0.12 ± 0.03	0.007	0.14 ± 0.03	0.15 ± 0.02	0.075
Dry weight, g	1750 ± 652	1872 ± 649	1312 ± 450	<0.001	1852 ± 657	1424 ± 526	0.004	1732 ± 705	1805 ± 475	0.626
Fat mass, g	135 ± 78	148 ± 81	91 ± 45	<0.001	148 ± 80	96 ± 54	0.004	159 ± 76	67 ± 25	<0.001
Free fat mass, g	1654 ± 675	1772 ± 673	1232 ± 499	0.001	1747 ± 681	1358 ± 574	0.013	1580 ± 705	1871 ± 536	0.058
Body volume, lt	1.75 ± 0.68	1.87 ± 0.68	1.29 ± 0.48	<0.001	1.85 ± 0.69	1.41 ± 0.56	0.006	1.70 ± 0.73	1.88 ± 0.53	0.264
Total body water, lt	1.37 ± 0.56	1.47 ± 0.56	1.01 ± 0.42	0.001	1.45 ± 0.57	1.12 ± 0.48	0.015	1.29 ± 0.58	1.59 ± 0.46	0.022
Intracellular water, lt	0.8 ± 0.8	0.9 ± 0.2	0.3 ± 0.1	0.503	0.9 ± 0.3	0.3 ± 0.1	0.497	0.9 ± 0.4	0.4 ± 0.1	0.586
Extracellular water, lt	0.9 ± 0.4	0.9 ± 0.4	0.6 ± 0.3	0.001	0.9 ± 0.4	0.7 ± 0.3	0.030	0.8 ± 0.4	1.0 ± 0.3	0.004
Extracellular mass, g	1169 ± 1097	1273 ± 1209	794 ± 334	0.070	1259 ± 1226	880 ± 387	0.141	1023 ± 451	1589 ± 1991	0.163
Body cell mass, g	585 ± 247	627 ± 250	437 ± 171	0.001	619 ± 254	478 ± 194	0.015	558 ± 258	665 ± 197	0.057
Skeletal muscle, g	439 ± 200	472 ± 205	322 ± 126	0.002	466 ± 208	352 ± 142	0.014	422 ± 214	487 ± 142	0.155
Total body minerals, g	43.8 ± 20.4	45.9 ± 21.1	36.2 ± 16.0	0.049	45.9 ± 21.0	37.1 ± 17.3	0.065	42.9 ± 21.7	46.4 ± 16.1	0.456
Total body mineral content, g	31.3 ± 14.9	32.7 ± 15.4	26.3 ± 11.8	0.076	32.7 ± 15.3	26.9 ± 12.8	0.099	30.5 ± 15.7	33.8 ± 12.1	0.334
Protein, kg	0.22 ± 0.09	0.23 ± 0.09	0.16 ± 0.06	0.001	0.23 ± 0.09	0.18 ± 0.07	0.012	0.21 ± 0.09	0.25 ± 0.07	0.061
Calcium, g	14.9 ± 7.1	15.5 ± 7.4	12.6 ± 5.6	0.085	15.5 ± 7.3	12.9 ± 6.1	0.114	14.4 ± 7.5	16.2 ± 5.8	0.281
Glycogen, g	9.8 ± 4.1	10.5 ± 4.2	7.3 ± 2.8	0.001	10.4 ± 4.2	8.0 ± 3.2	0.014	9.4 ± 4.3	11.1 ± 3.2	0.067

**Table A2:** Characteristics of the bioelectrical impedance analysis of the total and the subgroups of the cohort in neonates 32–37 weeks of gestational age.

	Total Cohort (*n* = 52)	Appropriate-for-Gestational Age (*n* = 40)	Small-for-Gestational Age (*n* = 12)	*p*	No Intrauterine Growth Restriction (*n* = 41)	Intrauterine Growth Restriction (*n* = 11)	*p*	Normal Fat Mass% (*n* = 35)	Decreased Fat Mass% (*n* = 17)	*p*
Fat mass, %	6.9 ± 3.3	7.1 ± 3.1	6.2 ± 4.0	0.429	7.1 ± 3.1	6.1 ± 3.9	0.392	8.6 ± 2.6	3.5 ± 0.9	<0.001
Fat-free mass, %	92.9 ± 3.2	92.7 ± 3.0	93.6 ± 3.9	0.454	92.7 ± 3.0	93.7 ± 3.9	0.365	91.3 ± 2.6	96.3 ± 0.7	<0.001
Total body water, %	77.2 ± 4.4	77.1 ± 4.2	77.3 ± 5.0	0.926	77.0 ± 4.3	77.9 ± 4.9	0.570	75.0 ± 3.4	81.7 ± 2.1	<0.001
Intracellular water, %	34.5 ± 6.0	34.2 ± 6.4	35.5 ± 4.6	0.546	34.9 ± 6.3	33.0 ± 5.0	0.349	35.1 ± 3.4	33.4 ± 9.5	0.347
Extracellular water, %	66.2 ± 4.1	66.7 ± 3.9	64.4 ± 4.6	0.100	66.0 ± 3.9	66.9 ± 5.0	0.516	65.0 ± 4.0	68.6 ± 3.5	0.003
Body density, g/cm^3^	1.02 ± 0.01	1.02 ± 0.01	1.02 ± 0.01	0.837	1.02 ± 0.01	1.02 ± 0.01	0.873	1.02 ± 0.01	1.03 ± 0.01	<0.001
Bone mineral density, g/cm^2^	0.25 ± 0.03	0.26 ± 0.02	0.22 ± 0.02	<0.001	0.26 ± 0.02	0.23 ± 0.03	0.003	0.25 ± 0.03	0.26 ± 0.02	0.819
Phase angle, degrees	8.1 ± 2.6	8.2 ± 2.7	7.7 ± 2.3	0.564	8.5 ± 2.8	6.6 ± 1.3	0.029	7.6 ± 1.9	9.1 ± 3.6	0.124
Impedance, ohms	711 ± 162	694 ± 146	769 ± 204	0.162	706 ± 154	731 ± 196	0.654	772 ± 153	586 ± 97	<0.001
Resistance, ohms	725 ± 124	720 ± 127	741 ± 118	0.617	716 ± 126	755 ± 115	0.362	742 ± 124	689 ± 119	0.153
Reactance, ohms	101 ± 28	97 ± 28	112 ± 28	0.134	100 ± 29	104 ± 25	0.691	97 ± 27	108 ± 31	0.186

**Table A3:** Neonatal outcomes of the total and the subgroups of the cohort in neonates 32–37 weeks of gestational age.

	Total Cohort (*n* = 52)	Appropriate-for-Gestational Age (*n* = 40)	Small-for-Gestational Age (*n* = 12)	*p*	No Intrauterine Growth Restriction (*n* = 41)	Intrauterine Growth Restriction (*n* = 11)	*p*	Normal Fat Mass% (*n* = 35)	Decreased Fat Mass% (*n* = 17)	*p*
Hypothermia	6 (11%)	4 (10%)	2 (17%)	0.612	5 (12%)	1 (9%)	0.999	2 (5%)	4 (23%)	0.081
Hypoglycemia	10 (19%)	6 (15%)	4 (33%)	0.212	6 (15%)	4 (36%)	0.190	3 (9%)	7 (41%)	0.009
Respiratory distress syndrome	9 (17%)	9 (22%)	0 (0%)	0.097	6 (15%)	3 (27%)	0.378	2 (5%)	7 (41%)	0.003
Patent ductus arteriosus	0 (0%)	0 (0%)	0 (0%)	n/a	0 (0%)	0 (0%)	n/a	0 (0%)	0 (0%)	n/a
Early-onset sepsis	4 (7%)	4 (10%)	0 (0%)	0.562	3 (7%)	1 (9%)	0.999	2 (5%)	2 (12%)	0.589
Late-onset sepsis	3 (6%)	2 (5%)	1 (8%)	0.999	3 (7%)	0 (0%)	0.592	2 (5%)	1 (6%)	0.999
Necrotizing enterocolitis	0 (0%)	0 (0%)	0 (0%)	n/a	0 (0%)	0 (0%)	n/a	0 (0%)	0 (0%)	n/a
Retinopathy of prematurity	0 (0%)	0 (0%)	0 (0%)	n/a	0 (0%)	0 (0%)	n/a	0 (0%)	0 (0%)	n/a
Intraventricular hemorrhage	1 (2%)	0 (0%)	1 (8%)	0.231	1 (2%)	0 (0%)	0.999	0 (0%)	1 (6%)	0.327
Bronchopulmonary dysplasia	0 (0%)	0 (0%)	0 (0%)	n/a	0 (0%)	0 (0%)	n/a	0 (0%)	0 (0%)	n/a
Length of hospital stay, days	14 (11–18)	13 (10–16)	17 (12–28)	0.040	13 (11–17)	17 (10–18)	0.661	12 (11–17)	14 (11–19)	0.417

**Table A4:** Characteristics of the bioelectrical impedance analysis of the total and the subgroups of the cohort in neonates <32 weeks of gestational age.

	Total Cohort (*n* = 49)	Appropriate-for-Gestational Age (*n* = 39)	Small-for-Gestational Age (*n* = 10)	*p*	No Intrauterine Growth Restriction (*n* = 36)	Intrauterine Growth Restriction (*n* = 13)	*p*	Normal Fat Mass% (*n* = 40)	Decreased Fat Mass% (*n* = 9)	*p*
Fat mass, %	9.1 ± 3.5	9.0 ± 3.7	9.5 ± 2.8	0.680	9.3 ± 3.5	8.3 ± 3.6	0.386	10.3 ± 2.5	3.4 ± 0.9	<0.001
Fat-free mass, %	90.8 ± 3.5	90.9 ± 3.6	90.4 ± 2.8	0.680	90.6 ± 3.4	91.6 ± 3.6	0.373	89.6 ± 2.5	96.3 ± 0.7	<0.001
Total body water, %	75.4 ± 4.7	75.6 ± 5.0	74.4 ± 3.4	0.480	75.2 ± 4.8	75.9 ± 4.7	0.653	73.6 ± 3.2	83.0 ± 1.9	<0.001
Intracellular water, %	37.1 ± 4.0	36.5 ± 4.2	39.5 ± 2.2	0.006	36.8 ± 4.1	38.0 ± 3.7	0.347	38.2 ± 3.2	32.1 ± 3.3	<0.001
Extracellular water, %	63.0 ± 4.2	63.7 ± 4.4	60.4 ± 2.2	0.003	63.4 ± 4.4	61.9 ± 3.7	0.273	61.9 ± 3.6	67.9 ± 3.3	<0.001
Body density, g/cm^3^	1.01 ± 0.01	1.01 ± 0.01	1.01 ± 0.01	0.648	1.01 ± 0.01	1.01 ± 0.01	0.304	1.01 ± 0.01	1.02 ± 0.01	<0.001
Bone mineral density, g/cm^2^	0.21 ± 0.02	0.22 ± 0.02	0.18 ± 0.02	<0.001	0.22 ± 0.02	0.20 ± 0.03	0.095	0.21 ± 0.02	0.25 ± 0.01	<0.001
Phase angle, degrees	9.2 ± 2.7	8.6 ± 1.8	11.5 ± 4.1	0.002	8.6 ± 2.2	10.9 ± 3.3	0.007	8.7 ± 2.3	11.3 ± 3.3	0.008
Impedance, ohms	953 ± 225	924 ± 226	1063 ± 195	0.084	950 ± 228	961 ± 226	0.879	1023 ± 184	640 ± 69	<0.001
Resistance, ohms	875 ± 195	873 ± 193	883 ± 214	0.894	871 ± 186	887 ± 228	0.799	912 ± 181	714 ± 182	0.005
Reactance, ohms	148 ± 69	152 ± 72	131 ± 58	0.399	146 ± 75	152 ± 54	0.810	150 ± 73	137 ± 53	0.602

**Table A5:** Neonatal outcomes of the total and the subgroups of the cohort in neonates <32 weeks of gestational age.

	Total Cohort (*n* = 49)	Appropriate-for-Gestational Age (*n* = 39)	Small-for-Gestational Age (*n* = 10)	*p*	No Intrauterine Growth Restriction (*n* = 36)	Intrauterine Growth Restriction (*n* = 13)	*p*	Normal Fat Mass% (*n* = 40)	Decreased Fat Mass% (*n* = 9)	*p*
Hypothermia	10 (20%)	7 (18%)	3 (30%)	0.663	6 (17%)	4 (31%)	0.422	5 (12%)	5 (55%)	0.011
Hypoglycemia	9 (18%)	8 (20%)	1 (10%)	0.663	5 (14%)	4 (31%)	0.220	4 (10%)	5 (56%)	0.006
Respiratory distress syndrome	26 (53%)	20 (51%)	6 (60%)	0.731	18 (50%)	8 (61%)	0.532	18 (45%)	8 (89%)	0.026
Patent ductus arteriosus	7 (14%)	5 (13%)	2 (20%)	0.620	4 (11%)	3 (23%)	0.363	7 (17%)	0 (0%)	0.322
Early-onset sepsis	3 (6%)	1 (3%)	2 (20%)	0.102	2 (5%)	1 (8%)	0.999	3 (7%)	0 (0%)	0.619
Late-onset sepsis	15 (30%)	10 (25%)	5 (50%)	0.247	10 (28%)	5 (38%)	0.500	14 (35%)	1 (11%)	0.242
Necrotizing enterocolitis	7 (14%)	3 (8%)	4 (40%)	0.025	5 (14%)	2 (15%)	0.999	7 (17%)	0 (0%)	0.322
Retinopathy of prematurity	4 (8%)	3 (8%)	1 (10%)	0.999	2 (5%)	2 (15%)	0.562	4 (10%)	0 (0%)	0.580
Intraventricular hemorrhage	10 (20%)	7 (18%)	3 (30%)	0.663	6 (16%)	4 (30%)	0.422	10 (25%)	0 (0%)	0.173
Bronchopulmonary dysplasia	13 (26%)	9 (23%)	4 (40%)	0.422	9 (25%)	4 (30%)	0.723	12 (30%)	1 (11%)	0.412
Length of hospital stay, days	45 (30–66)	44 (31–62)	52 (30–88)	0.517	44 (31–64)	47 (30–66)	0.675	30 (29–44)	48 (33–75)	0.047
